# Opponent processing in the retinal mosaic of nymphalid butterflies

**DOI:** 10.1098/rstb.2021.0275

**Published:** 2022-10-24

**Authors:** Primož Pirih, Marko Ilić, Andrej Meglič, Gregor Belušič

**Affiliations:** ^1^ Biotechnical Faculty, University of Ljubljana, Večna pot 111, 1000 Ljubljana, Slovenia; ^2^ Eye Hospital, University Medical Centre, Grablovičeva 46, 1000 Ljubljana, Slovenia

**Keywords:** vision, opponency, retina, photoreceptor, Nymphalidae

## Abstract

The eyes of nymphalid butterflies, investigated with incident illumination, show colourful facet reflection patterns—the eye shine—which is uniform or heterogeneous, dependent on the species. Facet colours suggest that the ommatidia contain different sets of photoreceptors and screening pigments, but how the colours and the cell characteristics are associated has not been clearly established. Here, we analyse the retinae of two nymphalids, *Apatura ilia*, which has a uniform eyeshine, and *Charaxes jasius,* a species with a heterogeneous eye shine, using single-cell recordings, spectroscopy and optical pupillometry. *Apatura* has UV-, blue- and green-sensitive photoreceptors, allocated into three ommatidial types. The UV- and blue-sensitive cells are long visual fibres (LVFs), receiving opponent input from the green-sensitive short visual fibres (SVFs). *Charaxes* has an expanded set of photoreceptors, allocated into three additional, red-reflecting ommatidial types. All red ommatidia contain green-sensitive LVFs, receiving opponent input from red receptors. In both species, the SVFs do not receive any opponent input. The simple retina of *Apatura* with three ommatidial types and two colour-opponent channels can support trichromatic vision. *Charaxes* has six ommatidial types and three colour-opponent channels. Its expanded receptor set can support tetrachromatic vision.

This article is part of the theme issue ‘Understanding colour vision: molecular, physiological, neuronal and behavioural studies in arthropods’.

## 1. Introduction

Colour vision is based upon the analysis of the spectral composition of the visual scene. The neural signals originating from photoreceptors with different spectral sensitivities are compared in a process of colour opponency. The spectral properties of colour opponent visual neurons match the statistics of natural scenes, maximizing the signal variance and allowing for optimal information processing in the visual pathway [1,2]. The optimal transformation for trichromatic vision is achieved through decomposing the retinal signals into an achromatic channel and two colour-opponent channels [3]. In flies and butterflies, the first stage of colour processing is performed already by the photoreceptors that directly inhibit each other through inter-photoreceptor synapses with histaminergic chloride channels [4–8]. Colour processing is continued downstream in the optical ganglia, the lamina and the medulla [5,8,9]. Here, we study colour opponency in the retinal mosaic of brush-footed butterflies (Nymphalidae).

Compound eyes are built from discrete optical units, the ommatidia. In all butterfly families with afocal apposition eyes studied thus far (families Papilionidae, Pieridae, Lycaenidae and Nymphalidae), each ommatidium contains eight large photoreceptors R1–8 and a small basal R9. The photoreceptors contribute their light-sensing microvilli to the common light guide, the rhabdom. The microvilli of R1,2 are oriented vertically (i.e. parallel to the dorsoventral eye axis), horizontally in R3,4 and diagonally in R5,7 and R6,8. The horizontal and diagonal photoreceptors R3,4 and R5–8 are the short visual fibres (SVF) that are presynaptic to the large monopolar neurons of the lamina [10,11], which in turn condition the signals for processing of achromatic contrasts and motion. In most butterflies studied so far, the SVFs are expressing a long-wavelength (LW) rhodopsin, peaking in the green range (520–560 nm), but exceptions do exist: in the dorsal eye of the lycaenid butterfly *Lycaena rubidus*, blue opsin mRNA is co-expressed with LW opsin mRNA in the females and exclusively expressed in the males [12].

The vertical photoreceptors R1,2 and the basal R9 are the long visual fibres (LVF) that project axons directly to the medulla and contribute to the processing of colour and polarization content of the visual scene. In most studied butterflies, receptors R1,2 express the short-wavelength (SW), ultraviolet (U, 340–370 nm) or blue-peaking (B, 420–460 nm) opsins [13,14]. The U and B receptors allocated to R1,2 give rise to three ommatidial types {UU, UB, BB} that are randomly distributed across the retina with species-specific fractions [15]. The random expression of the various visual pigments is driven by the *spineless* mechanism [16]. The basal cells R9 likely express the same LW opsin as R3–8 [13].

The retinae of papilionid and pierid butterflies are fully tiered [17]. The rhabdomeres of R1–4 occupy the distal tier of the rhabdom and the rhabdomeres R5–8 constitute the proximal tier. The SW part of the downwelling light is filtered by the visual and screening pigments in the distal tier, resulting in red-shifted sensitivities of the proximal photoreceptors R5–8 [18,19]. In papilionids, the basal LVF R9 can be either green- or red-sensitive [20] and expresses the LW opsin mRNA [21]. The red-sensitive basal LVF photoreceptor in pierids [11] likely also expresses a LW opsin. The inter-photoreceptor opponent synapses are located both downstream in the optic ganglia—the lamina and the medulla—and in the retina, along the photoreceptor axons, rendering the opponent signals detectable by intracellular recordings from the photoreceptors [5–7]. In the Japanese swallowtail, *Papilio xuthus*, opponent signals were detected in all photoreceptors R1–8 [6].

In brush-footed butterflies (nymphalids), the retina is incompletely tiered. The rhabdomeres of R3–8 are present along the entire length of the ommatidium, while the rhabdomeres of R1,2 may be present only distally [10,22] or may span along the whole rhabdom [13,23]. A tracheolar basket at the base of the retina forms the reflective *tapetum lucidum* in all studied butterfly families with apposition eyes [24,25] except in the papilionids [26]. The light launched into the rhabdom is reflected from the tapetum, bringing about *the eye shine*. The reflections from individual ommatidia are coloured, depending on the composition of visual pigments (rhodopsin R and its isomere metarhodopsin M) and the presence of screening pigments. A red ommatidial colour is a tell-tale sign for red (i.e. blue- and green-absorbing) screening pigment being opposed to the rhabdom. Ommatidia without such a screening pigment appear yellow, green, pale or blue, depending on the tuning of the tapetum, rhodopsin composition and rhabdom length [15,25,27,28]. In species with a uniform eye shine, e.g. *Vanessa atalanta*, vertical cells R1,2 express exclusively UV- or blue-peaking rhodopsins, similarly to papilionids and pierids [13,28].

Many nymphalids have a non-uniform eye shine with red-reflecting ommatidia [25,29], which may indicate the presence of more than three ommatidial types. For instance, in some *Heliconius* butterflies, R1,2 can express another type of UV opsin (UV2, due to opsin duplication) [14] or a green-absorbing (G; LW) opsin [30], which leads to the expansion of possible ommatidial types and a complex retinal mosaic. The LW opsin-expressing R1,2 likely reside in the red ommatidia, which furthermore contain a functional, red-sensitive R9 that inhibits the green-sensitive vertical cells [7]. The functional significance of R9 in the non-red ommatidia is currently unknown, but their spectral sensitivity is likely less red-shifted [15,28], similarly to *Papilio* [20].

Here, we study the inter-photoreceptor opponent mechanisms in brush-footed butterflies (family Nymphalidae). We focus our study on the lesser purple emperor *Apatura ilia*, and the two-tailed pasha, *Charaxes jasius*, the former with a simple and the latter with a complex eye mosaic. We show that the emperor is equipped with the basic trichromatic photoreceptor set, while the pasha has five additional photoreceptor types. We show that the expanded set of receptors in the pasha (green-sensitive R1,2, yellow-sensitive R3–9 and red-sensitive R9) is allocated to the red ommatidia. We identify two and three colour opponent channels in the emperor and the pasha, respectively. Taken together, the eyes of brush-footed butterflies can have either a simple retinal mosaic with three ommatidial types and two colour opponency channels, or a complex retinal mosaic with six ommatidial types and three opponency channels that are presumably used as the substrate for tetrachromatic vision.

## 2. Results

### (a) Eyeshine and rhodopsin photochemistry

**Figure 1.**
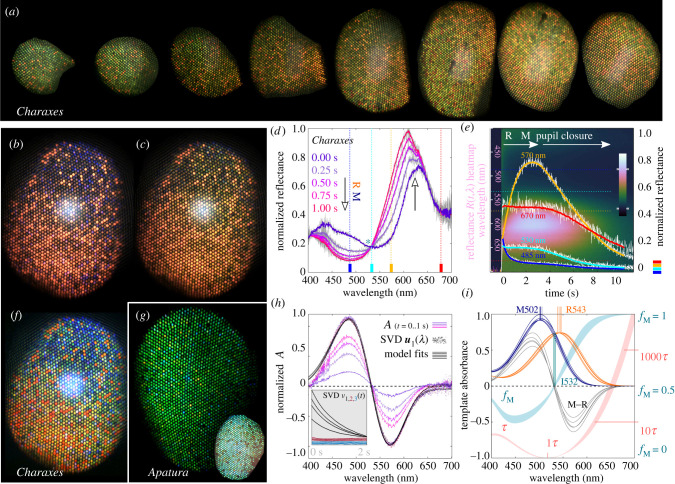
Eyeshine and spectroscopic determination of the LW opsin. (*a*) Images of the eyeshine in *Charaxes* with the eye rotated from dorsal to ventral in 15° angles (*left to right*). (*b,c,f*) The eyeshine of *Charaxes,* white-balanced images of (*b*) dark-adapted and (*c*) light-adapted state, (*f*) hyperspectral image of the dark-adapted state. (*g,j*) The eyeshine of *Apatura,* (*g*) hyperspectral image; (*j*) white-balanced image. (*d,e,h,i*) Spectroscopic determination of the main rhodopsin in *Charaxes*. (*d*) Reflectance spectra measured 0.25 s apart (five traces, *violet–magenta*); raw data as *grey traces*; isosbestic point,*asterisk;* (*e*) Filtered time traces of the eyeshine reflectance at 485, 530, 570 and 670 nm (*blue, cyan, orange and red traces*); raw data (*white* *traces*); normalized reflectance heatmap *R*(*t*, *λ*) in the background (400–700 nm); (*h*) normalized absorbance-difference Δ*A* between the dark-adapted spectrum (*t* = 0 s) and the spectra measured at *t* = [0.25, 0.50, 0.75, 1.00 s] (*violet–magenta traces*) from one experiment; singular value decomposition (SVD) spectral components ***u***_1_(*λ*) from five experiments on the same animal (*dots*); fitted template absorbance-difference models (*black traces*). Temporal SVD components ***v***_1-3_, ***v***_1_ exhibit an exponential decay with a similar time constant in four experiments (*inset, black traces*). (*i*) fitted absorbance-difference (M–R) spectra (*black traces*) from five experiments on one animal; estimated LW rhodopsin (*orange traces*) and metarhodopsin template absorbance spectra (*blue traces*); steady-state metarhodopsin fraction *f*_M_ (range 0–1, *cyan shade*), normalized isomerization time constant *τ*(*λ*) shown with a log *y*-axis (*red shade*).

The eye shine mosaic in *C. jasius* is predominantly green in the dorsal part and yellow-green in the central and ventral parts, speckled with red ommatidia whose fraction is higher ventrally (figure 1*a*). The red ommatidia stand out prominently in the hyperspectral image of the dark-adapted eye of *Charaxes* (figure 1*f*), while in the eye of *Apatura*, mosaic regionalization is not observed (figure 1*g,j*).

When the eye of *Charaxes* was left in the dark for longer than 10 min, the dorsal non-red ommatidia were blueish, turning green after a few seconds of illumination (figure 1*b*,*c*) before the pupil activation would extinguish the eyeshine. The reflectance change had two phases: the first phase was due to rhodopsin-metarhodopsin (R–M) photoisomerization [31]. Photoisomerization caused a pronounced reduction of reflectance below the isosbestic point at approximately 530 nm (figure 1*d*). The reflectance increase in the wavelength range (550…650 nm) is consistent with the observed eyeshine colour change (figure 1*b*,*c*). The second phase, due to the pupil closure [32], started about 3 s after the light onset and completed about 15 s afterwards (figure 1*e*).

The measured reflectance spectra were low-pass filtered, background-corrected and log-transformed. The difference between the first and any later log_10_(*R*) spectrum gave a series of absorbance-difference spectra Δ*A* with a peak–trough shape (figure 1*h*). The first 50 log_10_(*R*) spectra were processed using singular value decomposition (SVD) of the (wavelength × time) matrix. The principal temporal component ***ν***_1_(*t*) was decaying exponentially, as expected for a photoisomerization process. A fit of a template absorbance-difference model to the principal spectral component ***u***_1_(*λ*) yielded the estimate for *Charaxes* LW rhodopsin and metarhodopsin, peaking at 543 nm and 502 nm, respectively (R543/M502). The estimated R&M templates, the steady state metarhodopsin fraction *f*_M_(*λ*) and the normalized isomerization time constant *τ*(*λ*) for isoquantal monochromatic illumination are shown in figure 1*i*. The estimated LW opsin of *Apatura* is R527/M496 (electronic supplementary material, figure S1).

### (b) Intracellular recordings

**Figure 2.**
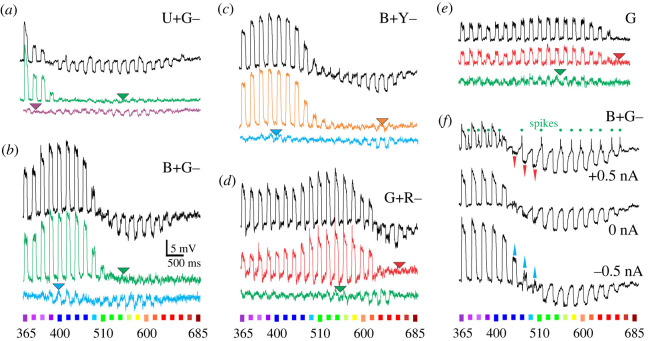
Receptor potentials of *Charaxes* photoreceptors, stimulated with a spectral sweep of light pulses. (*a–e*) Responses in dark-adapted state (black traces) and selectively adapted with steady monochromic light at wavelengths indicated with triangles (coloured traces); (*a–d*) opponent cells, (*e*) non-opponent cell. (*f*) Responses of a B+G– cell at resting membrane potential (0 nA), depolarized (+0.5 nA) and hyperpolarized (−0.5 nA); red and blue arrows indicate the reversal of opponent responses. Abbreviations in each panel indicate the cellular spectral class; colour bars at the bottom indicate stimulus (wavelength in nm). Adapting wavelengths: (*a*) 525 and 375 nm, (*b*) 525 and 400 nm, (*c*) 600 and 400 nm and (*d*,*e*) 625 and 525 nm.

Photoreceptor identities were revealed by intracellular recordings, using rapid spectral sequence stimulation provided by a fast narrow-band LED source [33]. In both species, we found receptors that depolarized maximally upon ultraviolet or blue stimulation, respectively, and hyperpolarized upon green stimulation (*Charaxes*: figure 2*a*,*b*; *Apatura*: electronic supplementary material, figure S2). We termed the two classes U+G− and B+G−; the letters signify the human colours of the unit spectral maxima, {UBGYR} for ultraviolet, blue, green, yellow and red, respectively. In *Charaxes*, we additionally found B+Y− photoreceptors and G+R− photoreceptors (figure 2*c*,*d*). The responses of the depolarizing units U+ and B+ could be isolated by saturating the hyperpolarizing units G− and Y− using appropriate LW adapting illumination (green or orange triangle, middle trace, figure 2*a–c*). Isolation of the hyperpolarizing units was less successful due to the sensitivity overlap with the depolarizing units in the SW range (purple or blue triangle, bottom trace, figure 2*a–c*). Both units of the G+R− opponent pair could be isolated by selective adaptation of the opponent unit (figure 2*d*), confirming that the sensitivity overlap between the G+ and R− units is minimal [7]. Hyperpolarization could be enhanced, suppressed or reversed by current injection (figure 2*f*). Both depolarizing and hyperpolarizing responses were graded along the approximately 3 log stimulus intensity range. The aperture of the light stimulus had a minor effect on the hyperpolarization in the green (electronic supplementary material, figure S2). The most frequently encountered cells belonged to the broad-sensitive LW photoreceptor class G and were without a detectable inhibitory input at the retinal level. In these cells, monochromatic adaptation light (of any wavelength) caused a wavelength-independent suppression of the light response (525 nm or 625 nm adapting light used in green and red traces in figure 2*e*).

**Figure 3.**
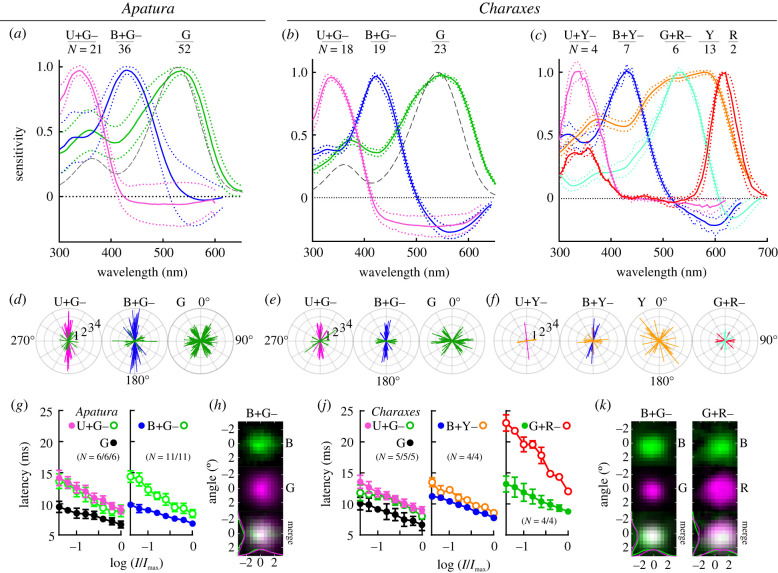
Electrophysiological analysis of photoreceptors. (*a–c*) Spectral sensitivity (mean ± s.e.m.; photoreceptor class and number of analysed cells indicated at the top; dashed grey curves, opsin templates with *λ*_max_ = (*a*) 527 nm and (*b*) 543 nm) in (*a*) *Apatura*; (*b*) *Charaxes*—basic set, (*c*) *Charaxes*—expanded set of photoreceptors. (*d*–*f*) Polarization sensitivity (PS) (bars indicate magnitude and angle in single cells; PS of opponent units indicated by green bars in U+G– and B+G–; orange bars in U+Y– and B+Y–; red bars in G+R–). (*g*,*j*) Response latency (mean ± s.d., number of analysed cells in brackets) of main cells (full circles), opponent units (empty circles) and non-opponent G cells (black circles) as a function of graded stimulus intensity. (*h*,*k*) Receptive fields of single main cells (top), their opponent units (middle) and overlap between main and opponent units (bottom). (*λ*_max_ and PS of all spectral classes given in table 1.)

The ancestral set of insect spectral photoreceptors {U, B, G} was found both in *Apatura* and *Charaxes* (figure 3*a*,*b*; electronic supplementary material, figure S2). The U and B receptors were maximally sensitive to vertically (parallel to the dorsoventral body axis) polarized light, consistent with allocation to photoreceptors R1,2. The cells of the main LW receptor class G were maximally sensitive either to horizontally or diagonally polarized light, consistent with the allocation to R3,4 and R5–8, respectively (figure 3*d,e*). In both species, the measured spectral sensitivity of G cells was broader and red shifted with respect to the corresponding opsin templates with peak sensitivity parameters (*λ*_max_) determined spectrophotometrically (*Apatura*: *λ*_max_ = 527 → 534 nm; *Charaxes*: *λ*_max_ 543 → 548 nm), probably due to self-screening in long photoreceptors [7,15,34]. In *Charaxes,* the additional, yellow-peaking LW receptor class Y (figure 3*c*) was similarly consistent with allocation to R3–8 (figure 3*f*). The G+R− receptors, allocated to R1,2, are maximally sensitive to green light and likely receive opponent input from the red-sensitive, basal R9 that could not be directly impaled [7].

**Table 1.** Spectral sensitivity maxima, polarization sensitivity and angular maxima of photoreceptor classes encountered in *Charaxes jasius* and *A. ilia*.

species	class	*λ*_max_	*λ*_max_ opp	PS	*N*	PS opp	*N*
*Charaxes jasius*	U+G−	337	500–590	1.7 ± 0.4	17	1.3 ± 0.5	6
	U+Y−	335	590	2.3	1	1.2 ± 0.1	3
	B+G−	430	560	1.7 ± 0.5	18	1.2 ± 0.1	7
	B+Y−	430	600	1.9 ± 0.5	6	1.3 ± 0.3	6
	G+R−	533	633	1.2 ± 0.1	6	1.3 ± 0.1	5
	G	548	—	1.8 ± 0.4	22	—	—
	Y	580	—	2.0 ± 0.4	12	—	—
	R	615	—	1.3 ± 0.1	5	—	—
*Apatura ilia*	U+G−	340	480–540	2.2 ± 0.6	20	1.3 ± 0.3	9
	B+G−	435	575	2.2 ± 0.6	44	1.2 ± 0.2	11
	G	534	—	2.2 ± 0.6	58	—	—

In *Charaxes*, the B cells with vertical microvilli were maximally hyperpolarized by their opponent units either around approximately 550 nm (figure 3*e*) or approximately 600 nm (figure 3*f*). The latter is best explained with the opponency of Y units to B cells, yielding the class B+Y− additional to the class B+G−. A similar, albeit more modest distinction could also be made between U+G− and U+Y− classes (figure 3*b,c*; electronic supplementary material, figure S3). The receptive fields of the main and the opponent units always overlapped, indicating that the opponency at the level of the retina does not involve pooling from neighbouring ommatidia (figure 3*h*,*k*).

Allocation of the main and the opponent units to the receptor positions was further studied by measuring the polarization sensitivity (PS) of the hyperpolarizing responses. In most UV+G− and some B+G− cells, PS in the green spectral range was low, suggesting that several green-sensitive cells converge onto a single UV or B cell, so their PS cancels out (figure 3*d*; electronic supplementary material, figure S3). In a subclass of B+G− and B+Y− cells, the opponent PS was modest (2–3) and had a horizontal angular maximum, while the main PS was modest to high (2–4): these opponent cells are good candidates for being the retinal substrate for polarization vision (figure 3*e;* electronic supplementary material, figure S3).

The onset of depolarizing responses was lagging the stimulus onset for 6–9 ms at the highest stimulus intensity and for 10–15 ms when being stimulated with light attenuated by 1.5 log (figure 3*g,j*). The shortest delay was, as expected, in the class G SVF photoreceptors and class Y photoreceptors (*not shown*); the fastest LVF was the class B*.* Response latency of hyperpolarizing units is a sum of the phototransduction and synaptic latency. In B+Y− cells, where the sensitivities of the main and opponent unit are about equal (electronic supplementary material, figure S4), the opponent response was delayed relative to the depolarizing response for approximately 1 ms, which is our estimate for the synaptic latency. This latency is consistent with the situation in U+G− class, where the latency of hyperpolarizing units G− was approximately 1 ms longer than that of SVF G cells (figure 3*g,j*). In this class, the opponent response was even slightly faster than the depolarizing response, possibly due to the slower phototransduction of the U+ unit. The most striking response delay difference was found in G+R− cells, where the opponent response was lagging the depolarizing response by 4–10 ms, likely due to the slow transduction in the minute, light-starved R9 cells (figure 3*j*; electronic supplementary material, figure S5).

### (c) Optical retinography

The electrophysiological results in *Apatura* suggested a simple retinal mosaic with two types of LVFs, both receiving opponent signals from G receptors. Physiological evidence suggests that the classes U+G− and B+G− are possibly allocated in pairs to form three types of ommatidia, {UU, UB, BB}, similarly to the genus *Vanessa* [15]. In the red-eyed *Charaxes*, however, the expanded retinal mosaic contains an additional distal LVF class G+R−, which could, in combination with U and B classes, form three additional ommatidial types, {GG, GU, GB}. The proposed allocation nevertheless awaits molecular validation in both species.

We checked the proposed allocation of six ommatidial types into the eye mosaic of *Charaxes* with optical retinography (ORG) [15], an optical method that reports the compound pupillary sensitivity of the photoreceptors in each ommatidium. We expected that the spectral sensitivity of the pupillary responses, evoked with isoquantal pulses, would resemble the weighted sum of opsin templates in LVFs and SVFs, with relative transduction gains as weights (the gain in cells {U,B} is about tenfold that of cells G; electronic supplementary material, figure S4). We note that the pupil is located distally in the retina, where the effects of screening and opponent signals are negligible. Additionally, the compound pupillary response should retain some PS in the non-red ommatidia in the UV–blue wavelength range, while the PS of red ommatidia that contain vertical LVF G photoreceptors would be very small (table 1).

**Figure 4.**
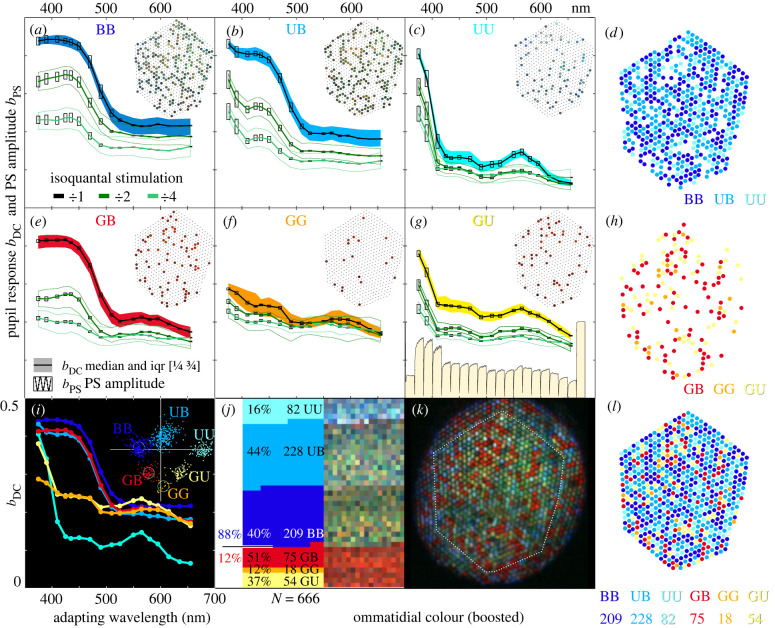
ORG of *C. jasius.* (*a,b,c,e,f,g*) Pupil sensitivity spectra of the six ommatidial clusters, BB (*a*), UB (*b*), UU (*c*), GB (*e*), GG (*f*) and GU (*g*), obtained with isoquantal adapting light (unattenuated: *black*, half intensity: *dark green*, quarter intensity: *light green*). The pupil responses expressed as reduction of ommatidial reflectance (0 = no response, 1 = maximal reduction obtained with a saturating broadband stimulus) are shown as the median (*solid lines*) and the inter-quartile range (*shaded areas*) of the constant pupillary responses *b*_DC_(*λ*). The median of pupil polarization sensitivity (modulation of the pupillary response, *b*_PS_(*λ*)) is shown with bars (figure 4*a*–*c*, *e*–*g*). Pupil responses from bouts 21−40 of the full intensity run are the traces at the bottom of (*g*). The pupil response *y*-axis range is [0.0 … 0.5]. Allocation of the types to the ommatidial lattice is shown in *insets.* (*d,h,l*) Allocation of the ommatidial types {BB,UB,UU} (*d*), types {GB,GG,GU} (*h*) and all six types (*l*) to the ommatidial lattice, cluster member counts at the bottom. (*i*) Plot of the pupil sensitivities of the six ommatidial clusters to unattenuated adapting stimuli; scatter plot of the principal two clustering scores of 666 ommatidia (*inset*). (*j*) fractions and counts of ommatidia in clusters (*left*), boosted ommatidial colours (*right*) obtained from the hyperspectral image. (*k*) Hyperspectral image of the dark-adapted ommatidial lattice.

We measured the compound spectral and PS of the individual ommatidial pupils in four male specimens of *Charaxes*. Here, we present the measurements from 666 ommatidia in the central eye region of a single eye. The pupil sensitivity was measured with an isoquantal spectral sequence (red to UV; UV to red). The sequence was repeated at full, half and quarter intensity. At each wavelength, we acquired bouts of 30 images: the first image was of the dark-adapted eye shine, the remaining were taken after 15 s adapting stimuli of linearly polarized light were delivered to the eye, causing a partial closure of the pupil. The polarizer was rotated for 37.25° between stimulations, completing three revolutions in 29 steps. For each ommatidium and each wavelength (*λ*) bout, we used a linear model to estimate the constant part of pupillary response *b*_DC_(*λ*) and its modulation part *b*_PS_(*λ*), which is due to the compound polarization sensitivity of photoreceptors. The constant (DC) and modulation (PS) parameters of the pupillary response were estimated using a linear model. The two parameters were normalized to the pupil working range, determined from the images taken in the dark-adapted state and in the fully light-adapted state. The two parameters were analysed with matrix singular value decomposition (SVD) and then classified using k-means clustering (see §4e). The six ommatidial clusters that formed (figure 4*i*) had compound pupillary spectral and polarization sensitivities consistent with the three basic ommatidial types {BB, UB, UU} and with the proposed ommatidial types containing green-sensitive LVFs {GB, GG, GU}.

**Figure 5.**
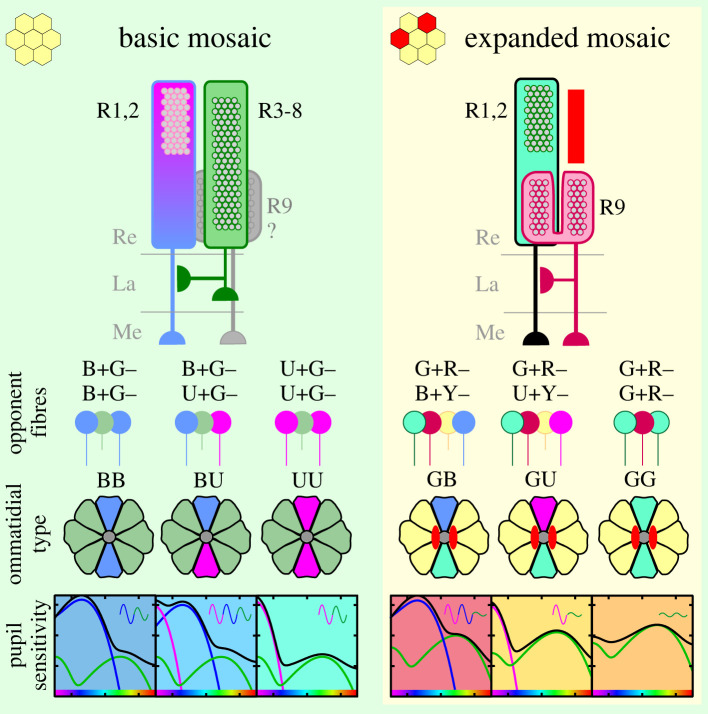
Proposed allocation of opponent photoreceptor pairs in the basic (*Apatura* and *Charaxes*) and the expanded (*Charaxes* only) retinal mosaic. Top, cellular identity of LVF (R1, 2, 9) and SVF (R3–8) photoreceptors in opponent pairs; middle, their allocation into ommatidial types (not yet confirmed with opsin mRNA or protein localization). U, B, G, Y, G+R– and R receptors are coloured pink, blue, green, yellow, teal and red, respectively; microvilli and rhabdoms are depicted with grey circles; red stripe above R9 and red ovals next to the rhabdoms indicate the red-screening pigment. Bottom, spectral sensitivity of pupil in each ommatidial type, a weighted sum of U, B and G LVF spectral sensitivities, superimposed on a background response of SVFs; the contribution of LVFs is scaled according to the transduction gain; the background is coloured as the ommatidial types in figure 4*a–g*. Sinusoidal insets indicate PS of the pupil in UV, blue and green (pink, blue and green curves, respectively); spectral range indicated with rainbows on the *x*-axis). Re, retina; La, lamina; Me, medulla.

The constant (DC) pupil responses of the ommatidial clusters *b*_DC_(*λ*) are shown as median and shaded inter-quartile range; the median of PS modulation *b*_PS_(*λ*) is depicted with bars (figure 4*a–c*,*e–g*). The clusters {BB,UB} had pronounced PS in the UV-blue spectral part (figure 4*a*,*b*). The cluster UU had a high PS across the whole spectrum (figure 4*c*). The remaining three clusters had generally lower PS (figure 4*e–g*). The distribution of the six clusters in the ommatidial lattice seems to be random (figure 4*d*,*h*). The boosted colours measured from the hyperspectral image of the eyeshine (figure 4*k*) were mapped to the six clusters. The two most numerous clusters {UB,BB} were green-shining, the cluster {UU} was blueish (figures 1*b* and 4*j*). Most notably, the three least numerous clusters with low PS (figure 4*e–g*) were all red-shining (figure 4*j*). The two more numerous red clusters are consistent with ommatidial types {GB,GU}; the least numerous cluster is likely of type GG (figure 5).

## 3. Discussion

We have shown that in *Apatura ilia*, a nymphalid butterfly with a basic set of spectral photoreceptors {U, B, G}, the retina is likely built from three ommatidial types without red pigments that form a simple mosaic. In *Charaxes jasius*, a nymphalid with an expanded set of LVF photoreceptor classes {G+R−, R} and a SVF photoreceptor subclass Y, the retinal mosaic is complex, having three additional ommatidial types, allocated to the red ommatidia (figure 5).

We found similar expanded spectral sets of photoreceptors in other species with red ommatidia and with a complex mosaic eye shine, e.g. the monarch (*Danaus plexippus*), blue morpho (*Morpho peleides*) and prepona (*Archaeoprepona demophon*) (electronic supplementary material, figure S6). Interestingly, these butterflies likely have only three opsin genes {U, B, G = LW} [35,36], suggesting that the expanded set of receptors {G+R−, Y and R} is implemented on the basis of optical filtering of a single LW opsin. The simple retinal mosaic can support trichromatic vision in the ultraviolet to green range [28,37]. Colour discrimination in the red wavelength range and the putative tetrachromatic vision are only supported in the eyes with a complex retinal mosaic, confirmed behaviourally in *Danaus* and *Heliconius* [28,38], but not yet in *Charaxes*. The retinal complexity is likely costly and seems to be evolutionarily switched on or off, depending on the visual ecology of the species. Extension of colour vision into the red wavelength range in brush-footed butterflies is associated with the simultaneous occurrence of multiple features of the visual system: the green-sensitive R1,2, the red-screening pigment, and a functional R9 with direct retinal opponency to the class G+R− LVF. These features are likely controlled by an additional stochastic genetic switch similar to the known *spineless* transcription factor [16].

Quite possibly, the expanded retinal mosaic is also associated with a red-shifted LW opsin. Nymphalid LW opsin is subjected to extensive evolutionary tuning, with *λ*_max_ varying between 515 nm and 565 nm [35], but the physiological relevance of this tuning is unknown. In nymphalids with a uniform eyeshine, the LW opsin tends to peak below 530 nm [27], whereas in the red-eyed butterflies, the LW opsin's peak tends to shift above 540 nm. A similar shift has been implicated in the evolution of red colour vision in lycaenid butterflies [39].

We note that a basal photoreceptor, receiving light filtered by red-screening pigments, can have high sensitivity and a high signal-to-noise ratio only by expressing a red-shifted LW opsin. Assuming narrow-band red light (620 nm), R9 would be approximately twice and five times more sensitive, if R525 is red shifted for 10 and 25 nm, respectively. Photoconversion of metarhodopsin seems to be very ineffective in a red-sensitive R9 (figure 1*i*), so the cell probably relies on enzymatic pigment conversion to maintain high sensitivity [40].

The simple nymphalid retina contains two colour-opponent channels (here U+G− and B+G−) and an achromatic channel (here SVF, G), following the design principles for optimal information transfer in trichromatic vision [3]. In the expanded retina, a new opponent channel (G+R−) and a Y subclass achromatic channel are added, likely on the basis of the same LW opsin as the green-sensitive R3–8. The channel expansion is implemented through a red-screening pigment that tunes the basal red receptor R9. Three interesting functional features can be elucidated. (i) LVF R1,2 can receive opponent inputs from either LVF or SVF (U&B from R3–8, G from R9). (ii) Opponency seems to be unidirectional, SVFs (R3-8) do not receive opponent inputs from LVFs. (iii) Opponent cells (G SVFs and R9 LVF) have a red-shifted sensitivity, compared to their postsynaptic partners R1,2. (We note that due to the absence of direct recordings, we cannot exclude the possibility that R9 are receiving an opponent input from another class of SVF.) This implementation is quite different from *Papilio*, where direct opponency is present among all kinds of visual fibres, including LVFs being opponent to both LVFs and SVFs, and SW-peaking classes being opponent to LW classes [5], or from *Drosophila*, where direct opponency is only present between the LVFs [4].

The SW photoreceptors U and B have a higher phototransduction gain (effectively more millivolts of depolarization per absorbed quanta) than the LW photoreceptors (electronic supplementary material, figure S4). Direct opponency from U&B LVF would cause strong inhibition of G&Y SVF. This would be detrimental for the signal-to-noise ratio in the achromatic visual pathway relayed via the lamina monopolar cells. The opponent signalling from SW to LW is likely implemented at a later stage in the visual pathway, probably via interneurons in the medulla [8,9]. In the complex nymphalid retina with red ommatidia, the tiny, light-starved, high-gain R9 cells can use a novel, *private opponent* channel in the form of green-sensitive LVF R1,2, thereby avoiding sending an opponent signal into the R3–8 achromatic pathway.

The LVFs in our study exhibited various levels of hyperpolarization due to the opponent cells; generally, the opponency in *Apatura* was much less pronounced than in *Charaxes* (figure 2*a–c*). The sensitivity of the receptors in opponent pairs must be tuned so that the degree of inhibition is *just right*. The inhibition should not be too weak, or else spectral discrimination would not work, nor too strong, to the detriment of signal generation and propagation. We hypothesize that the inhibition gain may be adjustable by yet unknown mechanisms that depend on light adaptation and internal state-dependent efferent inputs from the central nervous system. Interestingly, many LVFs from all spectral classes exhibited slow spikes (figure 2*f*; electronic supplementary material, figure S2*a*) that were very loosely correlated with light stimulation and depolarization. These spikes may have been initiated by the adjacent cells that may be modulating the physiological properties of their impaled neighbour. Further comparative research is needed to assess how the opponent pairs respond to the changes in spectral composition of ambient light across different time scales, and what are the optimal spectral opponent combinations for the particular photic environment.

## 4. Experimental methods

### (a) Animals

The butterflies were collected near Zadar and Mali Lošinj, Croatia (*C. jasius*) or bred and shipped from the UK (*A. ilia*) by Mr Mark Youles as a part of ongoing collaboration. Adult butterflies were kept at 27°C and 80% relative humidity and regularly fed sucrose solution.

### (b) Rhodopsin isomerization spectroscopy

The template spectra of the LW rhodopsin and metarhodopsin can be estimated using a spectroscopic method employing a broadband light source both for isomerization and the measurement [40]. The animal was immobilized in a pipette tip attached to a manual goniometer and placed under a Leitz Orthoplan microscope with a custom epi-illumination attachment with a 50% beamsplitter. The light source was a modified violet-chip-based white LED (Soraa MR16-50-B03) driven with a constant current source. A long working distance objective (either Nikon Plan ELWD 20 × NA0.40 1.2/160, part 120152, or an Olympus MPlan 10 × NA0.25 0/∞) was focussed on the eye's curvature centre. A Bertrand eyepiece was used to finely position the animal and set the illuminator's aperture and field iris so that the whole objective back-focal plane was filled with ommatidial reflections. The eye's luminous deep pseudopupil was imaged onto the iris plane of a custom-made microspectrometer head and relayed to a rosette-to-line fibre bundle attached to a spectrometer (Ocean Optics USB2000) controlled from GNU Octave. Eyeshine images were taken with a Raspberry Pi4, equipped with a Pi HQ Camera (RGB CMOS, 20MP).

Before each measurement, the animal was left to dark adapt for 10–60 min, so that the visual pigment would be predominantly in the rhodopsin isoform. The shutter was opened for 10–15 s, and several hundred spectra with integration time 15–25 ms were taken. Electronic (thermal) noise and the animal or objective background were subtracted from the spectral series. Data processing was implemented in GNU Octave with Signal and Optim toolboxes, using SVD, where a matrix with a temporal series of spectra **M** was decomposed into *n* spectral (**U**) and temporal (**V**) components, **M_[_***_λ_*_×t]_ → **U**_[*λ*×n]_**D**_[n×n]_**V_[_**_t×n]_^T^.

The raw data matrix **M**_0_ with count spectra (2048 pixels × 500 acquisitions) was decomposed into spectral and temporal components that were separately low-pass filtered using a zero-phase filter (function *filtfilt*). A filtered matrix **M**_1_ was recomposed from the first few signal-bearing components and then log-transformed, **M**_2_ = log_10_(**M**_1_). The first 50 spectra of **M**_2_ were again decomposed. The fundamental component {***u***_0_,***ν***_0_} was approximately constant in time and could be discarded, while the next temporal component vector ***ν***_1_ followed an exponential relaxation course, indicative of a photoisomerization process. The corresponding spectral component vector ***u***_1_, analogous to the absorbance-difference spectrum, was fitted with a template absorbance-difference model based on Govardovskii *et al.* [41] templates ***Γ****_λ_*, ***û***_1_ = *a* (*c**Γ**_M_ −* ***Γ****_R_*) + *ab,* where *c* is M/R peak absorbance ratio, *b* is (relative) baseline correction and *a* is a technical scaling parameter. We used nested models where the parameters could be constrained to *b* = 0 or *c* = 1.25, the latter being a biblical value for M/R peak absorbance ratio. A model fit yielded *λ*_*R*_, *λ*_*M*_ and optionally *c.* In the case of *Charaxes*, the value *c* = 1.25 seems to be correct, while the experimental data for *Apatura*, due to close-lying R&M peaks, does not allow for a reliable estimation of *c*. The data points below 430 nm were excluded from the fit due to a systematic deviation (see also electronic supplementary material, figure S1).

### (c) Electrophysiological recordings

Intracellular recordings were performed as described previously [7]. Briefly, immobilized animals were placed with the head in the centre of rotation into a goniometer that also carried the micromanipulator with sharp electrodes (Sensapex, Oulu, Finland). The recordings were performed with an amplifier (SEC-10LX, NPI, Tamm, Germany) in bridge mode or discontinuous clamp mode at 20 kHz and 0.25 duty cycle. The electrodes were pulled from borosilicate glass and had resistance in the range 80–120 MΩ when filled with 3 M KCl. Light stimulation was provided by a 75 W XBO lamp (Cairn Research, Kent, UK), filtered through a motorized monochromator (B&M Optik, Limburg, Germany), a computer-controlled neutral density wedge filter (Thorlabs, Dachau, Germany) and, for PS measurements, a UV-capable polarizer (OUV2500, Knight Optical, UK). The second source was a ‘LED Synth’ [33] with narrow-band LEDs between 365 and 685 nm in 15 nm intervals. Both sources were combined with a beam splitter (Thorlabs, Dachau, Germany) and isoquantized with an irradiance-calibrated spectrophotometer (Flame, Ocean Optics, USA) to yield equal photon (isoquantal) flux density at all wavelengths (max. 1.5 × 10^15^ photons cm^−2^ s^−1^). The aperture of the coaxial stimulating beam was adjusted to between 1.5° and 20° by an iris. Receptive fields of the photoreceptors were mapped with an RGB DLP projector (LightCrafter 4500, Texas Instruments, USA) that projected to a back-projection screen (ST-Pro-X, Screen-Tech e.K., Hohenaspe, Germany) at a refresh rate of 220 Hz using software package PsychoPy.

### (d) Eyeshine

Eyeshine was observed with a custom epi-illumination microscope built from Thorlabs, Edmund Optics and Linos parts as described elsewhere [15,29]. The relaying lenses were near-UV achromatic doublets (Edmund Optics). The main objective lens was a Zeiss LD-Epiplan 20 × NA0.40 objective (part 442840). Images were taken with monochrome or RGB CMOS cameras (1.6, 2.3, 20.0 MP, BFS-U3-16S2, BFLY-U3-23S6, BFS-U3-200S6, all FLIR/PointGray).

The illumination for RGB images was provided by a white LED (colour temperature approx. 3000 K), filtered by a purplish filter (Lee Filters) that brought the three colour channel gains close to unity. Hyperspectral images were taken with a monochrome camera, using the LED synth [33] as the light source. Exposure and gain were optimized for each image. The instrumental background, due to reflections from the objective lenses, was acquired separately, averaged and subtracted from the image series. Images were processed in ImageJ/Fiji [42]. Background correction was performed by subtracting an averaged image of lens reflections without the animal. The stacks were aligned using StackReg [43]. Substacks covering spectral ranges where a similar eyeshine pattern was observed (e.g. 380–440 nm, 480–540 nm and 600–660 nm) were averaged and joined into a pseudo-coloured image (figure 1*f* and figure 4*k*).

### (e) Optical retinography

ORG is an optical method reporting the compound sensitivity of the photoreceptors in each ommatidium [15]. We measured the pupillary action spectra of ommatidia in the central eye region of *Charaxes*. The experiments were performed in the epi-illumination microscope equipped with a monochrome CMOS camera as described in §4d. The light source for test images was a white LED, filtered with an orange- or red-coloured glass long-pass filter. The adapting light source was the LED synth [33] with 20 LEDs covering the range 365–660 nm. The combined beam was depolarized using a liquid crystal depolarizer (DPP25-A, Thorlabs) before being focussed to a 1 mm diameter, NA 0.40 polymer fibre. The fibre output was collimated with an aspheric lens and sent through a rotatable, UV-capable polarizer.

The adaptation experiment was conducted three times, at full, half and quarter intensity of the isoquantal adapting light source. In each of the experiments, we acquired *m* = 1200 images, divided into 40 bouts. In each bout, we recorded a dark-adapted image and 29 images where monochrome, isoquantal, linearly polarized adapting light was switched on for 15 s prior to taking the test image. The polarizer was rotated for 37.25° between the images, so the 30th image completed the third rotation in the same angular position as the second image. Each bout was followed by a 1 min dark adaptation period. The bouts #1 and #21 were dark-adapted control; the bouts #20 and #40 were control with saturating adaptation light. The bouts [#2 … #19] and [#22 … #39] were sequences with spectral adaptation going from red to UV and from UV to red, respectively. We reversed the bouts [#2 … #19], yielding six experimental runs with adaptation wavelength going from UV to red. An example of median responses of GU ommatidia from bouts 21–40 of the full intensity run is shown in figure 4*g*.

The ROI of ommatidia were found using ImageJ [42] functions ‘Find Maxima’ and ‘Analyse Particles’. The adaptation states of *n* = 666 ommatidia in *m* = 3600 images (6 experimental runs × 20 bouts × 30 images) were exported as a table of average ROI grey values *g*_[n__×__m]_. The pupil range of each ommatidium was obtained by finding the maximal grey value in the fully dark-adapted state *g*_n,DA_ and the minimal grey in the fully light-adapted state *g*_n,LA_. The relative pupil activation for the ommatidium *n* in image *m* was calculated as *p*_*n*,*m*_ = 1 − (*g*_*n*,*m*_ − *g*_*n*,LA_)/(*g*_*n*,DA_ − *g*_*n*,LA_).

The 30 pupil responses in each bout, ***p***_n,m_ = [*p*_*n*,m_ … *p*_*n*,*m*+30_]^T^ were fitted with a linear model ***p*** = **A*b***. The matrix **A**_[30×5]_ was constructed from column vectors containing a constant (DC) component, sine and cosine modulation, linear trend and exponential decay with a predetermined time constant. Solving the matrix equation ***b* *=* A*\p*** for each bout yielded a parameter vector containing the constant pupillary response parameter *b*_DC_ and the oscillating parameters {*b*_cos_, *b*_sin_}. From the latter two, PS modulation amplitude was calculated as *b*_PS_ = 2|(*b*_cos_ + î*b*_sin_)|; we did not analyse the PS phase. The parameters *b*_DC_ and *b*_PS_ obtained at each wavelength in the six experimental runs were placed into six **B**_DC_ and six **B**_PS_ matrices of size [666 ommatidia × 18 wavelengths]. Each of these 12 matrices were separately normalized and decomposed using SVD, yielding score and spectral matrices. The first three score vectors [666 × 3] from each decomposition were concatenated, yielding a matrix of size [666 ommatidia × 36 scores], coming from two parameters {*b*_DC_, *b*_PS_} × 6 experimental runs × 3 score vectors. This matrix was again decomposed, giving the final score matrix with 5–6 signal-bearing score vectors that were used for k-means clustering. The first two-score components are shown as the scatter in figure 4*i inset*.

## Data Availability

Data are available from the Dryad Digital Repository: https://doi.org/10.5061/dryad.9cnp5hqkq [44]. The data are provided in the electronic supplementary material [45].
